# Factors Affecting Turnover Intention among New Graduate Nurses: Focusing on Job Stress and Sleep Disturbance

**DOI:** 10.3390/healthcare10061122

**Published:** 2022-06-16

**Authors:** Minjeong An, Seongkum Heo, Yoon Young Hwang, JinShil Kim, Yeonhu Lee

**Affiliations:** 1 College of Nursing, Chonnam National University, Gwangju 61469, Korea; anminjeong@jnu.ac.kr; 2 Georgia Baptist College of Nursing, Mercer University, Atlanta, GA 30341, USA; heo_s@mercer.edu; 3 Seoul Women’s College of Nursing, Seoul 03617, Korea; hyy0926@snjc.ac.kr; 4 College of Nursing, Gachon University, Incheon 21936, Korea; kimj503@gachon.ac.kr; 5 Department of Trauma Ward, Chonnam National University Hospital, Gwangju 61469, Korea

**Keywords:** turnover intention, job stress, sleep disturbance, generation Z, new nurse

## Abstract

Despite the high prevalence of nurses’ turnover and the turnover intention of new nurses, there are insufficient studies examining turnover intention at the time when job orientation is completed and independent nursing commences. Thus, this study examined turnover intention levels and identified the factors affecting turnover intention of new Generation Z nurses, focusing on job stress and sleep disturbance, at the eighth week after completing job orientation. This was a cross-sectional descriptive correlational study. Using a convenient sampling method, 133 new nurses were recruited. Data were collected using a structured questionnaire consisting of demographic and occupational characteristics, job stress, sleep disturbance, and turnover intention. Descriptive statistics were computed to describe the sample and interest variables. Logistic regression analysis was performed to examine the association of job stress and sleep disturbance with turnover intention. Most nurses were women (91.7%) and approximately two-thirds worked in the surgical ward (n = 61, 45.9%). Turnover intention was 12.8%, average job stress was 40.11 ± 90.7, and average sleep disturbance was 42.39 ± 15.27. New graduate nurses’ turnover intention was associated with job stress (OR = 1.07, 95% CI = 1.02–1.12) and sleep disturbance (OR = 1.19, 95% CI = 1.05–1.35), and this model explained 47.7% of the variance. Study findings determine that job stress and sleep disturbance were significant predictors of turnover intention in new nurses at the eighth week after joining the hospital. Therefore, nursing administrators should focus on new nurses’ job stress and sleep disturbance, and provide them with timely assessment and management to reduce turnover intention.

## 1. Introduction

In medical institutions, nurses play an important role in providing quality of care, which is critical for good patient outcomes [1]. To provide high quality of care and improve patient outcomes, it is critical to maintain an adequate nurse-to-patient ratio. However, the global nursing shortage is estimated to be 5.9 million, which is even more evident in the context of COVID-19 [2]. In addition, one out of six nurses is expected to retire within 10 years, and the number of new nurses required to replace the retirees is estimated to be at 4.7 million [2]. In South Korea, although the number of licensed nurses is higher than that of those in the Organisation for Economic Co-Operation and Development (OECD) countries (19.2 vs. 14.8 per 1000 population), the number of nurses in clinical settings was lower than that in other OECD countries (5.9 vs. 9.1 per 1000 population) [3], indicating a nursing shortage in South Korea. Furthermore, high rates of nurse turnover have increased this shortage in several countries.

In the United States, nurses’ hospital turnover rates from 2016 to 2020 increased by 2.8%, which resulted in an 18.7% turnover rate in 2020 [4]. In South Korea, nurses’ overall turnover rate between 2012 and 2016 (49.7%) at acute care hospitals was considerably higher than of those in the United States [5]. In the study, the risk for turnover in new nurses who worked less than one year was double than that in nurses with more than five years of work experience. Among new nurses, turnover rates within the first and second six months of working was 20.1% and 6.3%, respectively [6]. More importantly, according to the Hospital Nurses Association, the turnover rates in new nurses increased from 15.4% in 2006 to 45.5% in 2018, illustrating a 30% increase during a 12-year period [7]. High nurse turnover rates increase nursing shortages, which can lead to poor patient outcomes and high health costs.

Nurse turnover deteriorates the activities of daily living, worsens existing pressure ulcers, or causes the development of new pressure ulcers, and increases 30-day hospitalization rates, medical errors, and the possibilities of receiving infection control citations in the United States and South Korea [8,9,10,11]. In addition, high nurse turnover rates considerably increase costs. In the United States, the average cost of a registered nurse’s turnover is more than USD 40,000, which results in an average hospital cost of more than USD 5 million per year [4]. Considering the high turnover rates in new nurses, and the negative consequences on patient outcomes and health costs, turnover in new nurses must be reduced.

Turnover intention is one factor affecting actual turnover. Turnover intention refers to the thoughts and behaviors among organizational members to voluntarily leave the organization [12]. In a study [13], turnover intention among 491 female nurses was 23%, and it was significantly associated with actual turnover within 12 months. In the study, 20% of nurses who had turnover intention resigned from their job within the next 12 months, while only 8% of nurses who did not have turnover intention left their job. In a meta-analysis [14], the pooled turnover intention was approximately 28% among nurses in 23 countries, which was similar to that in South Korea. Turnover intention in new nurses was also prevalent (34%) [15]. Therefore, nurse turnover intention must be decreased to reduce actual turnover.

According to the review findings of some turnover intention theories [16], a variety of factors were included (e.g., motivation factors that contribute to job satisfaction, and hygiene factors that contribute to job dissatisfaction [17]). To decrease turnover intention, it is critical to determine factors affecting turnover intention in nurses. For example, sleep disturbance and job stress can affect nurse turnover intention [18,19,20].

Although many studies have been conducted on the relationship between job stress and sleep quality or the sleep disturbance of nurses and nursing students [21,22], there are few studies reporting the relationship between these two factors with turnover intention. However, some studies reported the relationship between turnover intention and each job stress or sleep problem. In nurses with varied work experience, higher levels of sleep disturbance were significantly associated with higher levels of turnover intention [20,23]. In another study with nurses in their first two years of working [24], higher levels of sleep disturbance was significantly associated with actual turnover. Nurses often experience sleep disturbance due to shift work [25]. In particular, new nurses can experience improved or worsened sleep disturbance because of changes in life patterns related to shift work, and having to care for other people with inexperienced work skills and a lack of self-regulation ability [26]. Sleep disturbance can increase turnover intention or actual turnover by decreasing nurses’ ability to cope with stress and by weakening their work resilience [24,26].

In a systematic literature review [18], job stress in new nurses was significantly related to turnover intention. In the review, most new nurse samples had six months to one year of work experience, and in new nurses, job stress at the beginning was the highest and gradually reduced over time [18,27]. The level of job stress in new nurses was the highest during the first month, and the level of turnover intention was the highest between the first and second months [28]. These findings are critical, considering the major characteristics of new nurses who are hired. To illustrate, most new nurses belong to Generation Z, which does not prefer overtime and weekend work [29]. However, this is difficult to avoid due to shift work.

Despite the high prevalence of turnover and turnover intention, and higher levels of sleep disturbance and job stress in new nurses, the associational relationships of these factors with turnover intention have not been frequently examined. In addition, considering that the intention of new nurses is still high, and the timing at which new nurses’ turnover intention occurs is becoming faster, it is necessary to examine their turnover intentions and the related factors at the end of job orientation, which is an earlier time after joining the hospital. Therefore, the purpose of this study was to determine the levels of turnover intention, and to identify the relationships of sleep disturbance, health status, and job stress with turnover intention in new nurses who had eight weeks of work experience, controlling for subjective health status and department (surgical vs. intensive care unit (ICU)). Subjective health status was selected as a covariate because, in nurses with varied work experience, poor health status was significantly associated with higher levels of turnover intention [30]. The department was selected as a covariate because ward type was significantly associated with turnover in nurses [10]. With this purpose, two hypotheses are proposed:

**Hypothesis** **1.** *Job stress is positively associated with turnover intention among new nurses working at a tertiary hospital.*

**Hypothesis** **2.** *Sleep disturbance is positively associated with turnover intention among new nurses working at a tertiary hospital.*

## 2. Methods

### 2.1. Design, Sample, and Data Collection

This was a cross-sectional, descriptive, correlational study that examined turnover intention at eight weeks among new graduate nurses who began working at their first hospital. The participants were recruited from a tertiary general hospital with 1050 beds located in the city of Gwangju, South Korea. The inclusion criteria of this study were new graduating nurses who had eight weeks of work experience (one week of job orientation at the hospital level and seven weeks of orientation at the ward or department level) and who agreed to participate in this study. The nurses worked under the supervision of their preceptors during their orientation in the ward, but were scheduled to work independently from the ninth week onwards. The exclusion criterion was nurses who joined the hospital through new career recruitment.

Using a convenience sampling method, the participants were recruited from 7 September 2020 to 20 September 2021. The participants then completed the pen-and-paper questionnaire.

### 2.2. Measurements

Job stress was measured using the Korean Occupational Stress Scale (KOSS-SF) [31]. The KOSS-SF consisted of 24 items measured on a 4-point Likert scale. The scale has seven subdomains: job demands (4 items), job autonomy (4 items), relationship conflict (3 items), job instability (2 items), organizational system (4 items), inadequate compensation (3 items), and workplace culture (4 items). The total score of each subdomain was converted according to the method suggested by the developer of the scale. The possible total score ranged from 24 to 96. Higher scores indicated higher job stress. Validity was supported by both content and construct validity, and reliability was supported by a Cronbach’s α of 0.82 in a prior study (Chang et al., 2005), and by a Cronbach’s α of 0.82 in this study.

Sleep disturbance was measured using the General Sleep Disturbance Scale that was developed by Lee (1992) [32] and translated into Korean by Choi et al. (2012) [33]. The scale consists of 21 items measured on an 8-point Likert scale (0–7 points), and assesses sleep problems in the past week. The scale has six subdomains: sleep initiation (1 item), sleep maintenance (2 items), sleep quality (3 items), amount of sleep (2 items), daytime function (7 items), and use of sleep induction methods such as drugs (6 items). Possible total scores ranged from 0 to 147. Higher scores indicated a greater degree of sleep disturbance. Validity was supported by concurrent validity [34], and reliability was supported by a Cronbach’s α of 0.75 in the study of Choi et al. [33], and a Cronbach’s α of 0.83 in this study.

Turnover intention was measured using a single item question (“Are you currently considering changing your job?”) [35]. It was dichotomized as “yes” with responses ranging from “agree” to “strongly agree”, and as “no” with responses ranging from “disagree” to “strongly disagree” on a 4-point Likert scale.

### 2.3. Data Analysis

The data were analyzed using SPSS/WIN 25.0 software. Descriptive statistics, including mean and standard deviation, were used to describe sample characteristics, including levels of turnover intention. The independent t-test and chi-squared test were used to examine the differences in sample characteristics, job stress, and sleep disturbance according to turnover intention. Multivariable logistic regression was used to determine the associations of sleep disturbance and job stress with turnover intention, controlling for subjective health status and work unit, and the suitability of the regression model was confirmed using the Hosmer–Lemeshow test.

## 3. Results

### 3.1. Sample Characteristics

The average age of the sample (N = 133) was 23.17 years old (Table 1). Most nurses were women (91.7%) and had earned a bachelor’s degree (86.7%). The nurses worked at different departments, namely, a surgical ward (45.9%), internal medicine ward (38.3%), and ICU (15.8%). Most nurses (74.4%) rated their health status as not bad or not good. The preferred shift type was day with 63 (48.5%), followed by evening with 52 (40.4%), and night with 15 (11.5%).

### 3.2. Levels of Turnover Intention and Differences Based on Sample Characteristics

The turnover intention of new nurses was 12.8% (n = 17). Nurses who had turnover intentions had a bad subjective health status (1.7% vs. 17.6%; χ^2^ = 10.40, *p* = 0.025) and worked in the ICU (12.1% vs. 41.2%; χ^2^ = 10.12, *p* = 0.009).

### 3.3. Difference in Turnover Intention Based on Job Stress and Sleep Disturbance: Bivariate Analyses

The average job stress in the sample was 40.11 ± 9.07 points (Table 2). The average job stress in nurses with turnover intention was significantly higher than that of their counterparts (47.55 ± 8.45 vs. 38.98 ± 8.65; t = −3.89, *p* < 0.001). Subdomains showing higher job stress in the nurses with turnover intention than their counterparts were job demand (t = −2.15, *p* = 0.033), interpersonal conflict (t = −2.30, *p* = 0.023), and lack of reward (t = −5.84, *p* < 0.001).

The average total score of sleep disturbance in the sample was 42.39 ± 15.27. The average total score in nurses without turnover intention was significantly higher than that in their counterparts (57.59 ± 16.83 vs. 40.16 ± 13.75 points; t = −4.74, *p* < 0.001). Subdomains showing significantly higher sleep disturbance in nurses with turnover intention than that of their counterparts were difficulty getting to sleep (t = −3.54, *p* = 0.001), waking up during sleep (t = −4.02, *p* < 0.001), quality of sleep (t = −2.35, *p* = 0.020), and fatigue and alertness at work (t = −3.13, *p* = 0.006).

### 3.4. Association of Job Stress and Sleep Disturbance on Turnover Intention

Logistic regression analysis was performed to examine the associations of job stress and sleep disturbance on turnover intention, controlling for subjective health status and department (Table 3). Model 1 included only health status and department as possible factors associated with turnover intention, and both variables were significantly associated with turnover intention and explained 20.3% of the variance (χ^2^ = 15.25, *p* = 0.004, and Nagelkerke R^2^ = 0.203). The nurses who had rated their subjective health status as “bad” had a higher odds ratio of turnover intention than that of their counterparts (OR = 15.56, 95% CI, 1.60–151.61; *p* = 0.018). The nurses who worked in the ICU had a higher odds ratio of turnover intention than that of their counterparts (OR = 4.09, 95% CI, 1.11–15.11; *p* = 0.035). In Model 2, job stress and sleep disturbance were added, and job stress and sleep disturbance with the department were significantly associated with turnover intention (H1 and H2 were accepted) and explained 47.7% of the variance (χ^2^ = 23.87, *p* < 0.001, and Nagelkerke R^2^ = 0.477). The turnover intention in new nurses increased by 7% and 19% with a one score increase in job stress (OR = 1.07, 95% CI, 1.02–1.12; *p* = 0.006) and sleep disturbance (OR = 1.19, 95% CI, 1.05–1.35; *p* = 0.007), respectively. Turnover intention in new nurses considerably increased among nurses working in the ICU (OR = 10.12, 95% CI, 1.68–60.99; *p* = 0.012).

## 4. Discussion

The findings of this study demonstrate that a considerable proportion of new nurses who have just completed their central and departmental training for eight weeks had turnover intentions. Furthermore, job stress and sleep disturbance were important factors associated with their turnover intention. Although the average scores in the sample were not very high, higher levels of job stress and sleep disturbance still increased the likelihood of turnover intention. These findings suggest that the assessment and management of job stress and sleep disturbance should be systematically conducted from the early stage of nurse employment to reduce turnover intention. In particular, assessment and management should be provided for new nurses working in the ICU. The findings of this study provide valuable information regarding the major targets of interventions to reduce turnover intention, and, in turn, actual turnover and the possible timeline inbetween.

The turnover intention of new nurses with eight weeks of work experience was 12.8%, which was lower than a pooled turnover intention of 28% among nurses with varied work experience from 23 countries and 31% in new nurses with the first three months of work experience [14,28]. In addition to the time difference (three months vs. two months) and country (Taiwan vs. South Korea) between the study of Yeh et al. (2009) and this study, there were differences in sample characteristics and work environment, including gender (100% vs. 91.7% women), educational level (26% vs. 86.7% bachelor’s degree), level of working independence (60% vs. 0%), and nurses working at several hospitals vs. one hospital. However, in the study of Yeh et al. (2009), education level and working independence were not associated with turnover intention. Furthermore, the turnover intention of new nurses who were going to work independently in this study was 12.8%. This value may have mainly been due to the pressure and responsibility on nurses to take the initiative in caring for patients alone. In several studies [13,36], turnover intention was significantly associated with actual turnover within the next 12 months after the training period. Therefore, turnover intention must be assessed and managed from the training period of new nurses to reduce actual turnover later on.

In prior studies [37,38], new nurses had higher job stress than that of experienced nurses due to difficulties in interpersonal relationships and a lack of work skills when being exposed to a new environment and learning new work. In addition, new Generation Z nurses were assumed to have higher levels of job stress. This is because Generation Z nurses are “digital natives” who have been exposed to the Internet and digital culture since childhood; thus, their relationship-forming skills are not mature [39]. However, in this study, the level of job stress in the new nurses was relatively low (item mean score, 1.7 points out of 1–4) and similar to the level in 447 nurses with varied work experience, including 3.1% of new nurses (1.8 points) [40]. Comparatively, the level of job stress was lower than the level (2.3–2.6 points) in nurses with varied work experience, including 37.1% of nurses with less than five years of experience [20]. However, in this study, job stress was significantly associated with turnover intention, which was consistent with the findings in prior studies showing the significant relationship between job stress and turnover intention in nurses with varied work experience, including new nurses [18,20,27,41]. These findings suggest the important role of job stress in turnover intention regardless of the level of work experience. Therefore, job stress must be assessed and managed in nurses, regardless of work experience, to reduce turnover intention.

Nurses can experience sleep disturbance due to irregular life patterns, including shift work. In particular, new nurses can experience more sleep disturbance than experienced nurses do due to sudden changes in sleep patterns [25] and frequent overtime work due to a lack of skills [20,26]. However, in this study, sleep disturbance in new nurses was relatively mild (42.4 points out of 0–147), and was lower than the level in nurses with varied work experience (31.8 points out of 15–60: translated score: 55.0 points out of 0–147) [20]. Despite the mild level of sleep disturbance in new nurses, sleep disturbance was significantly associated with turnover intention in this study, which was consistent with the findings in prior studies of nurses with varied work experience [20,23]. The findings of prior studies and this study demonstrate the important role of sleep disturbance in turnover intention regardless of the level of nurses’ work experience. Therefore, sleep disturbance must be assessed and managed in all nurses, including new nurses.

In this study, the level of turnover intention in new nurses who worked in the ICU was significantly higher than their counterparts. In a prior study [20], nurses who worked in the ICU had significantly higher levels of job stress (71.3) than those who worked in other units (63.4–64.5). Nurses working in ICUs may have higher levels of job stress than that of those who work in other units because of continuous monitoring, reporting, and/or caring for more severe patients, requiring specialized knowledge, skills, and clinical judgment. Findings from a prior study and this study imply that nurses working in the ICU have higher levels of turnover intention due to higher levels of job stress. Therefore, turnover intention and job stress in nurses working in the ICU must be assessed and managed, regardless of work experience, as early as possible.

According to the findings of this study and prior studies, turnover intention in all nurses, regardless of work experience, must be reduced by effectively managing job stress and sleep disturbance. Reducing turnover intention is critical because turnover intention affects actual turnover within one year [13]. In this study and in a prior study [42], turnover intention due to three types of job stress, namely, job demand, interpersonal conflict, and lack of reward, was significantly different in new nurses. Comparatively, job stress affecting turnover intention in experienced nurses was due to inadequate compensation and workplace culture [20]. These findings imply that interventions targeting a reduction in turnover intention need to consider nurses’ work experience.

## 5. Limitations

This study has several limitations. First, a convenient sampling method from only one tertiary hospital and the small sample size may limit the generalizability of the study’s findings. Second, this was a cross-sectional study; thus, caution in the interpretations of the study’s findings that infer causality must be taken. Therefore, further studies should be conducted with a probabilistic sampling method, a larger sample, and utilizing a longitudinal study design. In addition, researchers need to examine how the job stress and sleep disturbance of new nurses are changed in the trajectories from the time of joining the new nurse to one year later, and how these two variables and turnover intention are associated in the trajectory.

## 6. Conclusions

In this study, the turnover intention of new nurses was examined after they had been employed at the hospital and had received eight weeks of orientation. At that point, their turnover intention was 12.8%, which is a value that should not be overlooked. Job stress and sleep disturbance, known as factors influencing turnover intention of new nurses, were still identified as significant influencing factors, even at the eighth week of employment. Therefore, nursing administrators should focus on the job stress and sleep disturbance of new nurses, and provide them with timely assessment and management to reduce turnover intention.

## Figures and Tables

**Table 1:** Participants’ demographic and occupational characteristics (*N* = 133).

Characteristics	Categories	Total	Turnover Intention			
		Mean ± SD or n (%)	No (n = 116)	Yes (n = 17)	x^2^	*p*
			n (%)			
Age (year)		23.17 ± 1.40			0.76	0.385
	<23	98 (73.7)	84 (72.4)	14 (82.4)		
	≥24	35 (26.3)	32 (27.6)	3 (17.6)		
Gender	Man	11 (8.3)	11 (9.5)	0 (0)	1.76	0.185
	Woman	122 (91.7)	105 (90.5)	17 (100)		
Marital status	Single	132 (99.2)	116 (100)	16 (94.1)	6.88	0.128
	Married	1 (0.8)	0 (0)	1 (5.9)		
Education level	Associate degree	16 (12.5)	14 (12.6)	2 (11.8)	0.17	1.000
	Bachelor’s degree	111 (86.7)	96 (86.5)	15 (88.5)		
	≥Master’s degree	1 (0.8)	1 (0.9)	0 (0)		
Subjective health status	Bad	29 (21.8)	2 (1.7)	3 (17.6)	10.40	0.025
	Not bad/Not good	99 (74.4)	88 (75.9)	11 (64.7)		
	Good	5 (3.8)	26 (22.4)	3 (17.6)		
Preferred work shift type	Day	63 (48.5)	54 (47.8)	9 (52.9)	1.26	0.492
	Evening	52 (40.0)	47 (41.6)	5 (29.4)		
	Night	15 (11.5)	12 (10.6)	3 (17.6)		
Department	Medical	51 (38.3)	45 (38.8)	6 (35.3)	10.12	0.009
	Surgical	61 (45.9)	57 (49.1)	4 (23.5)		
	Intensive care unit	21 (15.8)	14 (12.1)	7 (41.2)		

**Table 2:** Participants’ job stress and sleep disturbance (*N* = 133).

Characteristics	Categories	Total	Turnover Intention			
		Mean ± SD	No (n = 116)	Yes (n = 17)	t	*p*
Job stress	Overall	40.11 ± 9.07	38.98 ± 8.65	47.55 ± 8.45	−3.89	<0.001
	Job demand	62.25 ± 14.28	61.23 ± 13.83	69.12 ± 15.80	−2.15	0.033
	Insufficient job control	49.37 ± 12.64	48.84 ± 12.14	52.94 ± 15.57	−1.25	0.213
	Interpersonal conflict	27.40 ± 14.12	26.34 ± 13.63	34.64 ± 15.66	−2.30	0.023
	Job insecurity	24.69 ± 19.20	23.71 ± 18.41	31.37 ± 23.48	−1.55	0.125
	Organizational system	36.74 ± 11.09	36.23 ± 10.93	40.20 ± 11.87	−1.38	0.170
	Lack of reward	45.79 ± 14.92	43.19 ± 13.31	63.40 ± 13.47	−5.84	<0.001
	Occupational climate	34.15 ± 16.05	33.12 ± 15.79	41.18 ± 16.53	−1.95	0.053
Sleep disturbance	Overall	42.39 ± 15.27	40.16 ± 13.75	57.59 ± 16.83	−4.74	<0.001
	Difficulty getting to sleep	2.43 ± 2.05	2.20 ± 1.92	4.00 ± 2.26	−3.54	0.001
	Waking up during sleep	4.57 ± 3.50	4.13 ± 3.26	7.59 ± 3.69	−4.02	<0.001
	Quality of sleep	13.47 ± 4.51	13.12 ± 4.55	15.82 ± 3.43	−2.35	0.020
	Quantity of sleep	4.48 ± 1.89	4.40 ± 1.86	5.06 ± 2.01	−1.35	0.178
	Fatigue and alertness at work	16.90 ± 7.69	15.89 ± 6.78	23.76 ± 10.04	−3.13	0.006
	Use of substances to help induce sleep	0.54 ± 1.30	0.42 ± 1.07	1.37 ± 2.23	−1.71	0.105

**Table 3:** Predictors of turnover intention among new graduate nurses working at a tertiary hospital (*N* = 133).

Predictors	Model 1				Model 2			
	b	OR	95% CI	*p*	b	OR	95% CI	*p*
Constant	−0.50	0.61		0.370	13.90	0.00		<0.001
Subjective health status, bad	2.74	15.56	1.60−151.61	0.018	0.82	2.27	0.16−33.19	0.549
Subjective health status, NGNB	0.06	1.06	0.26−4.33	0.931	−1.39	0.25	0.45−1.37	0.110
Department, surgical	−0.67	0.51	0.13−2.06	0.346	−0.76	0.47	0.10−2.26	0.344
Department, ICU	1.41	4.09	1.11−15.11	0.035	2.31	10.12	1.68−60.99	0.012
Job stress					0.07	1.07	1.02−1.12	0.006
Sleep disturbance					0.17	1.19	1.05−1.35	0.007
x^2^ (p)	15.25 (0.004)				23.87 (<0.001)			
Hosmer–Lemeshow test	0.791				0.124			
Nagelkerke R2	0.203				0.477			

Reference: health status, good; department, medical; Abbreviations: CI, confidence interval; ICU, intensive care unit; NGNB, not good and not bad.

## Data Availability

The data presented in this study are available on request from the corresponding author. The data are not publicly available because it was not approved from the Institutional Review Board.
